# Lack of alignment between orthopaedic surgeon priorities and patient expectations in total joint arthroplasty

**DOI:** 10.1186/s13037-023-00365-w

**Published:** 2023-06-29

**Authors:** Harsh N. Shah, Andrew A. Barrett, Patrick H. Le, Prerna Arora, Robin N. Kamal, Derek F. Amanatullah

**Affiliations:** 1 School of Medicine, Mayo Clinic, 200 1st Street SW, Rochester, MN 55905 USA; 2 Department of Orthopaedic Surgery, University of Pittsburg Medical Center - Hamot, 201 State Street, PA Erie, 16550 USA; 3 Department of Orthopaedic Surgery, Stanford University, School of Medicine, 450 Broadway Street, Redwood City, CA 94063 USA

**Keywords:** Total joint arthroplasty, Patient expectations, Patient-centered care, Surgeon priorities

## Abstract

**Background:**

Healthcare systems are shifting toward “patient-centered” care often without assessing the values important to patients. Analogously, the interests of the patient may be disparate with physician interests, as pay-for-performance models become common. The purpose of the study was to determine which medical preferences are essential for patients during their surgical care.

**Methods:**

This prospective, observational study surveyed 102 patients who had undergone a primary knee replacement and/or hip replacement surgery about hypothetical scenarios regarding their surgical experience. Data analysis included categorical variables presented as a number and percent, while continuous variables presented as mean and standard deviation. Statistical analysis for anticoagulation data included the Pearson chi-square test and one-way ANOVA test.

**Results:**

A large majority, 73 patients (72%), would not pay to have a four-centimeter or smaller incision. The remaining 29 patients (28%) would prefer to have a four-centimeter or smaller incision and would pay a mean of $1,328 ± 1,629 for that day. A significant number of patients preferred not to use anticoagulation (*p* = 0.019); however, the value attributed to avoiding a specific method of anticoagulation was found not to be significant (*p* = 0.507).

**Conclusions:**

The study determined the metrics prioritized by hospitals and surgeons are not important to the majority of patients when they evaluate their own care. These disconnects in the entitlements patients expect and receive can be solved by including patients in discussions with physicians and hospital systems.

**Supplementary Information:**

The online version contains supplementary material available at 10.1186/s13037-023-00365-w.

## Background

Healthcare systems are shifting toward “patient-centered” care often without specifically assessing the values and preferences of actual patients [1]. At the same time, the interests of the patient may be disparate with the interests of the physician, as pay-for-performance models become increasingly common [1]. Much research has been conducted in an attempt to quantify which aspects of post-surgical care can be optimized [1, 2]. For example, there is a recent growth in minimally invasive surgery (MIS) in total hip and total knee arthroplasty [2]. Procedures such as the direct anterior approach to total hip arthroplasty and the Oxford technique in knee replacement have gained popularity due to their tissue sparing benefits [3, 4]. To meet patient demands, hospitals introduce ever increasing new instruments, surgical tables, robots, and components to capitalize on the growing trend of MIS [4]. Telemedicine is also emergent technology bringing healthcare to patients who are immobile or reside far from their physician [5]. Robb et al. highlighted cost and time savings from implementation of such a program [5]. From a physician perspective, telemedicine can provide a convenient way to observe patients in their home environment [5]. However, other patient populations may not perceive telemedicine as advantageous [6]. For example, geriatric patients were less likely to be satisfied with telemedicine software [6]. Still, a survey study by Khairat et al. indicated telemedicine as an “effective tool for receiving follow-up care, with no differences in mean satisfaction between age categories” [6]. As physician workload increases, healthcare systems continually seek efficiency to delivering care [1, 6].

Trying to achieve patient-centered care, while at the same time focusing on decreasing costs to the healthcare system, has the potential to create conflict between opposing incentives of the patient and the healthcare system delivering their care [1, 6]. We sought to elucidate which aspects of care patients prefer. Furthermore, we will attempt to quantify or at least compare which medical preferences are relatively worthwhile for patients. These preferences include properties such as incision length, hospital length-of-stay, postoperative experiences, follow-up personnel, and type of anti-coagulation. Improving healthcare quality must include the patient as a stakeholder directly.

## Methods

This prospective observational study used an institutional review board approved, consented survey (Supplemental Figure 1) of patients after total knee arthroplasty or total hip arthroplasty to evaluate and quantitate their pre-, peri-, intra-, postoperative preferences. We collected the variables age, sex, and socioeconomic elements (e.g., race, health insurance, years of education, work status).

### Participants

A total of 102 participants completed the survey, of which 43% underwent a total knee replacement, 6% underwent a partial knee replacement, 43% underwent a total hip replacement, 6% underwent a total knee and total hip replacement, and 2% underwent a partial knee and total hip replacement (Table 1).

**Table 1:** Patient demographics

Patient demographics	Response (*n* = 102)
Age (years)	64.9 ± 13.2
Race	
White/Caucasian	66 (64%)
Black or African-American	2 (2%)
American Indian or Alaska Native	0 (0%)
Asian	14 (14%)
Hispanic	12 (12%)
Native Hawaiian or other Pacific Islander	1 (1%)
Other or none specified	6 (6%)
Income	
Less than $15,000	29 (28%)
$15,000 to $29,999	14 (14%)
$30,000 to $49,999	10 (10%)
$50,000 to $99,999	18 (18%)
$100,000 to $249,999	6 (6%)
No answer given	3 (3%)
Employment Status	
Full-time employed	23 (23%)
Part-time employed	5 (5%)
Retired	45 (44%)
No work outside the home	2 (2%)
Disabled	14 (14%)
Unemployed	9 (9%)
No answer given	4 (4%)
Education Level	
Elementary School	6 (6%)
High School	31 (30%)
2-year college degree	22 (22%)
4-year college degree	26 (26%)
Post-college graduate degree	17 (17%)
Relationship Status	
Married	57 (56%)
Single, never married	9 (9%)
Divorced/separated	20 (20%)
Widowed	16 (16%)
Insurance Status	
Medicaid	12 (12%)
Medicare	64 (63%)
Health insurance from employer	34 (33%)
Health insurance purchased out of pocket	3 (3%)

### Statistics

Categorical variables presented as number and percent. Continuous variables presented as mean and standard deviation. Data to determine difference between expected and observed frequencies for the route of anticoagulants were statistically evaluated with the Pearson chi-square test (GraphPad Prism, version 6). Data for variance analysis for the route of anticoagulant were statistically evaluated with the one-way ANOVA test (GraphPad Prism, version 6). Two-tailed *p*-values of < 0.05 were considered significant.

## Results

### Incision length

**Fig. 1:**
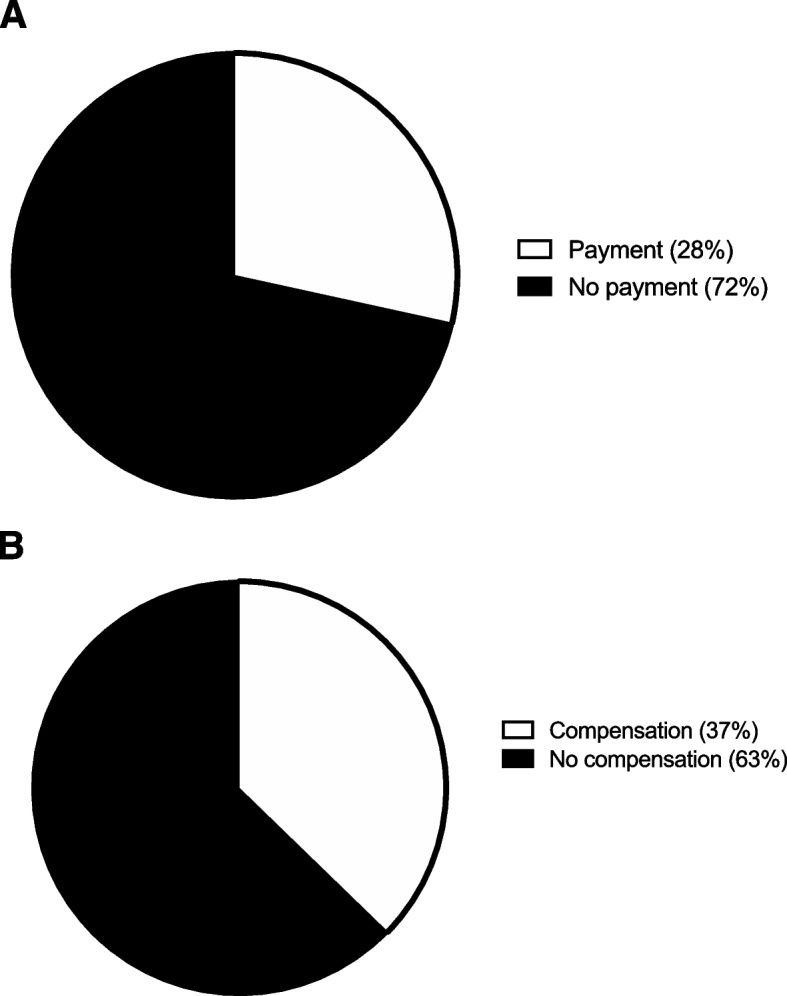
Incision length. **A** The percentage of participants indicating their willingness to pay to have a 4 cm smaller incision. **B** The percentage of participants indicating their willingness to be compensated to have a 4 cm larger incision

A large majority, 73 participants (72%), would not pay to have a 4 cm or smaller incision. The remaining the 29 patients (28%) who preferred to have a 4 cm or smaller incision would pay as much as $1,328 ± 1,629 (Fig. 1A). A majority, 64 participants (63%), did not want to be compensated to have a 4 cm or larger incision. However, 38 remaining patients (37%) wanted to be compensated as much as $2,771 ± 2,257 for a 4 cm or larger incision (Fig. 1B).

### Duration of hospital stay

**Fig. 2:**
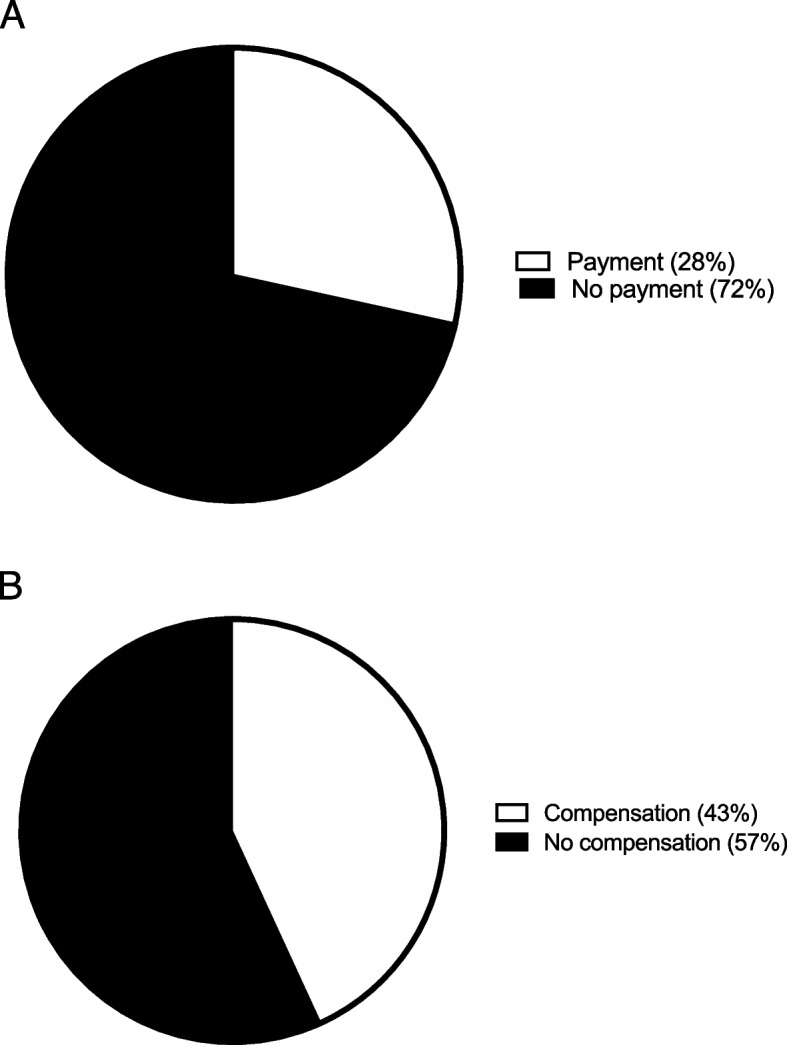
Duration of hospital stay. **A** The percentage of participants wanting to pay in order to extend their hospital stay by one day. **B** The percentage of participants needing compensation to shorten their hospital stay by one day

A large majority, 73 participants (72%), would not pay to extend their hospital stay by one day. However, the remaining 29 patients (28%) who would prefer to stay one day longer would pay a mean $827 ± 1,291 for that day (Fig. 2A). A majority, 58 patients (57%), would not need to be compensated to have their hospital stay shortened by one day. However, 44 patients (43%) would need to be compensated a mean $2,569 ± 2,253 to shorten their hospital stay by one day (Fig. 2B).

### Returning to activity

**Fig. 3:**
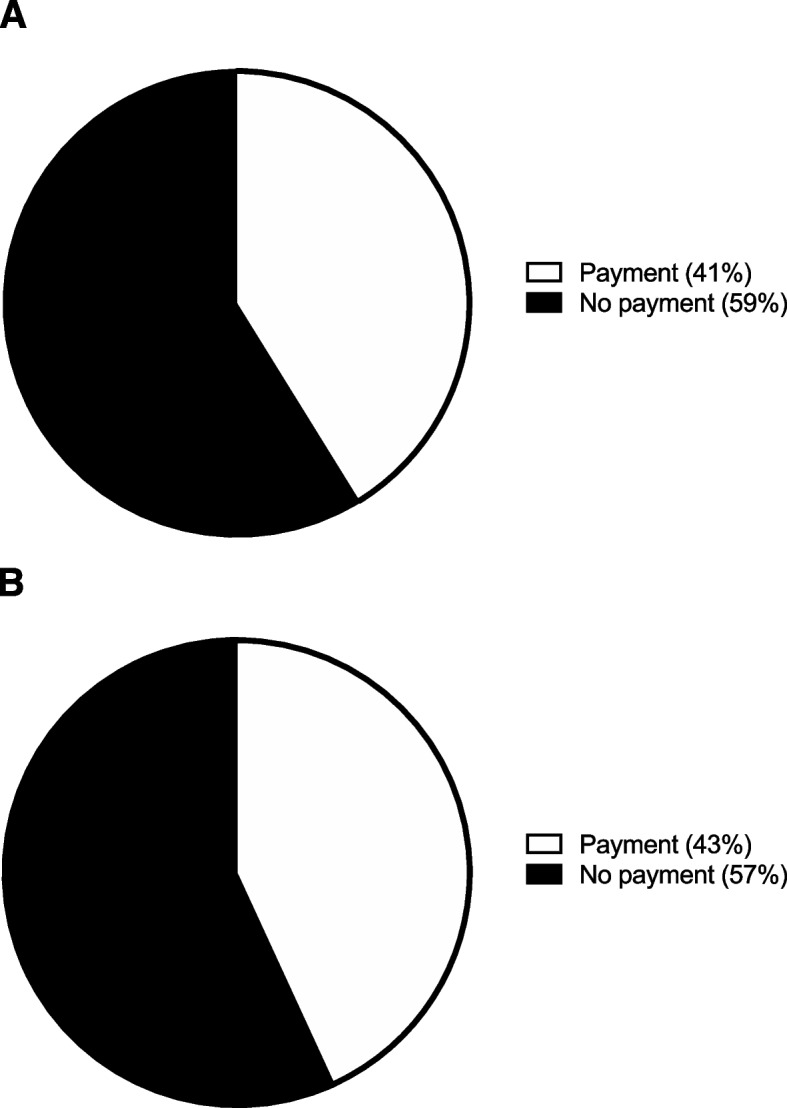
Returning to activity. **A** The percentage of participants indicating their willingness to pay to return to normal activity two weeks sooner. **B** The percentage of participants indicating their willingness to pay to return to normal activity four weeks sooner

A majority, 60 patients (59%), would not pay to return to normal activity two weeks sooner. However, 42 patients (41%) would pay a mean of $1,695 ± 1,845 to return to normal activity two weeks sooner (Fig. 3A). A majority, 58 patients (57%), would not pay to return to normal activity four weeks sooner. However, 44 patients (43%) would pay a mean of $1,502 ± 1,675 to return to normal activity four weeks sooner (Fig. 3B).

### Implants

**Fig. 4:**
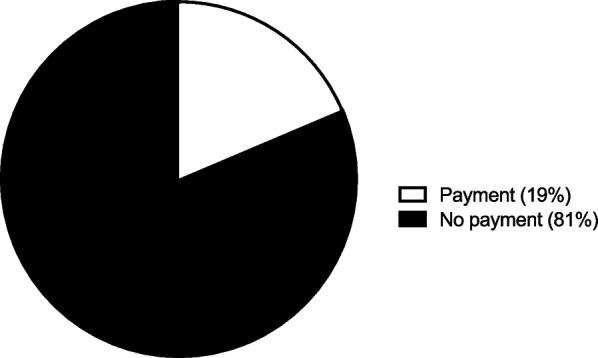
Implants. The percentage of participants indicating their willingness to pay to return to choose their own implants

A large majority, 83 patients (81%), would not pay to choose their own implants. However, 19 patients (19%) would pay a mean $985 ± 1,445 to choose their own implants instead of having the physician choose them (Fig. 4).

### Discharge disposition

**Fig. 5:**
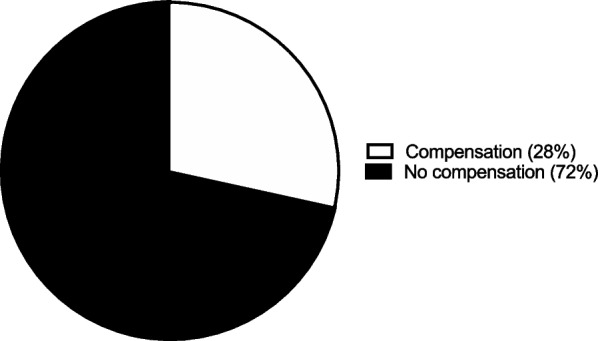
Assistance from home health aide. The percentage of participants requiring compensation to forego help from a home health aide

**Fig. 6:**
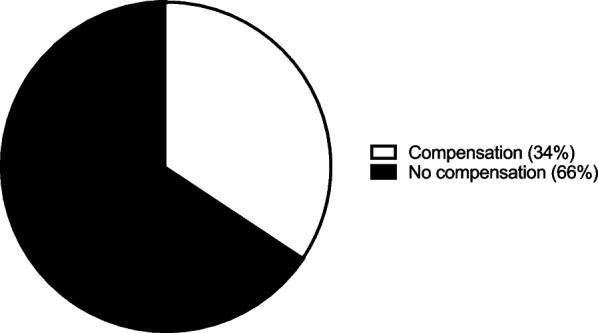
Physical therapy. The percentage of participants expecting compensation to forego physical therapy

**Fig. 7:**
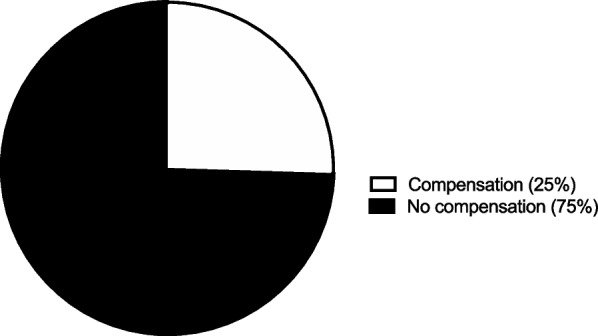
Skilled nursing facility. The percentage of participants needing compensation to forego a skilled nursing facility

A large majority, 73 patients (72%), would not need to be compensated to forego the help of a home health aide. However, the remaining 29 patients (28%) would need to be compensated a mean $849 ± 1,227 to forego the help of a home health aide (Fig. 5). Additionally, a majority 67 patients (66%) would not need to be compensated to forego physical therapy. However, 35 patients (34%) would need to be compensated a mean $2,220 ± 1,981 to forego physical therapy (Fig. 6). Furthermore, a large majority, 76 patients (75%), would not need to be compensated forego a skilled nursing facility. However, 26 patients (25%) would need to be compensated a mean $2,559 ± 2,378 to return home after surgery instead of to a skilled nursing facility (Fig.7).

### Follow-up

**Fig. 8:**
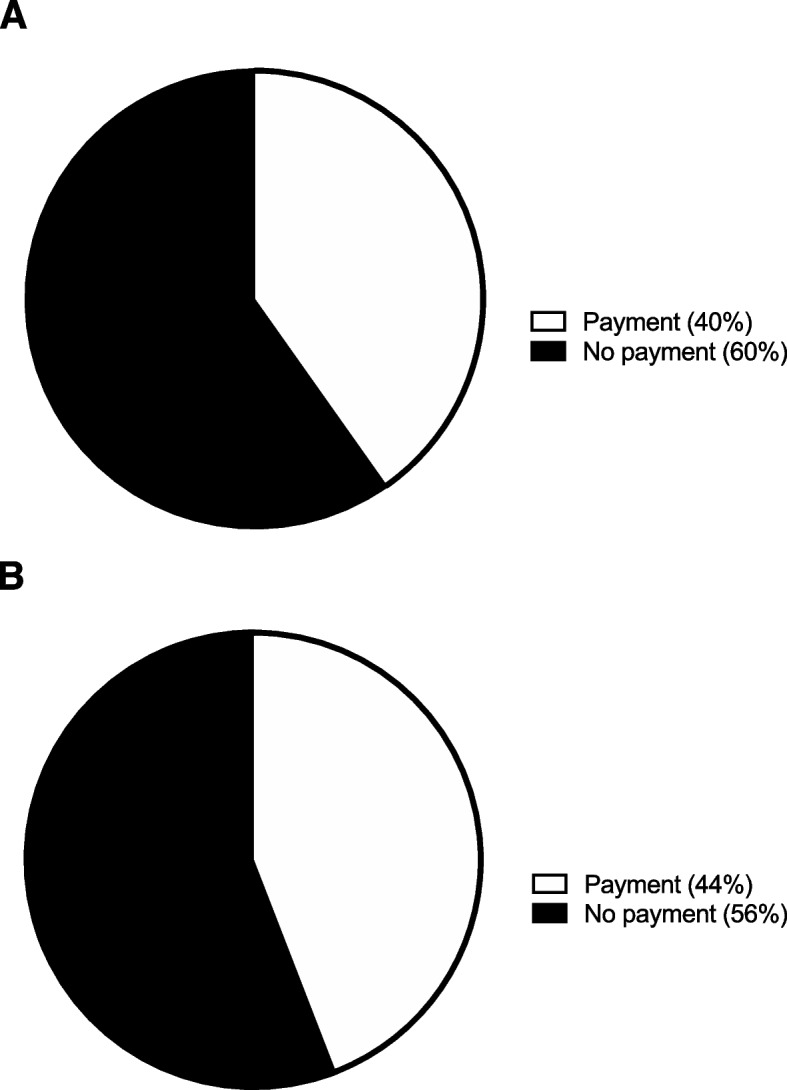
Follow-up with a provider other than the physician. **A** The percentage of participants indicating their willingness to pay for a follow-up with their physician rather than another provider at the two-week follow-up visit. **B** The percentage of participants indicating their willingness to pay for a follow-up with their physician rather than another provider at the six-week follow-up visit

**Fig. 9:**
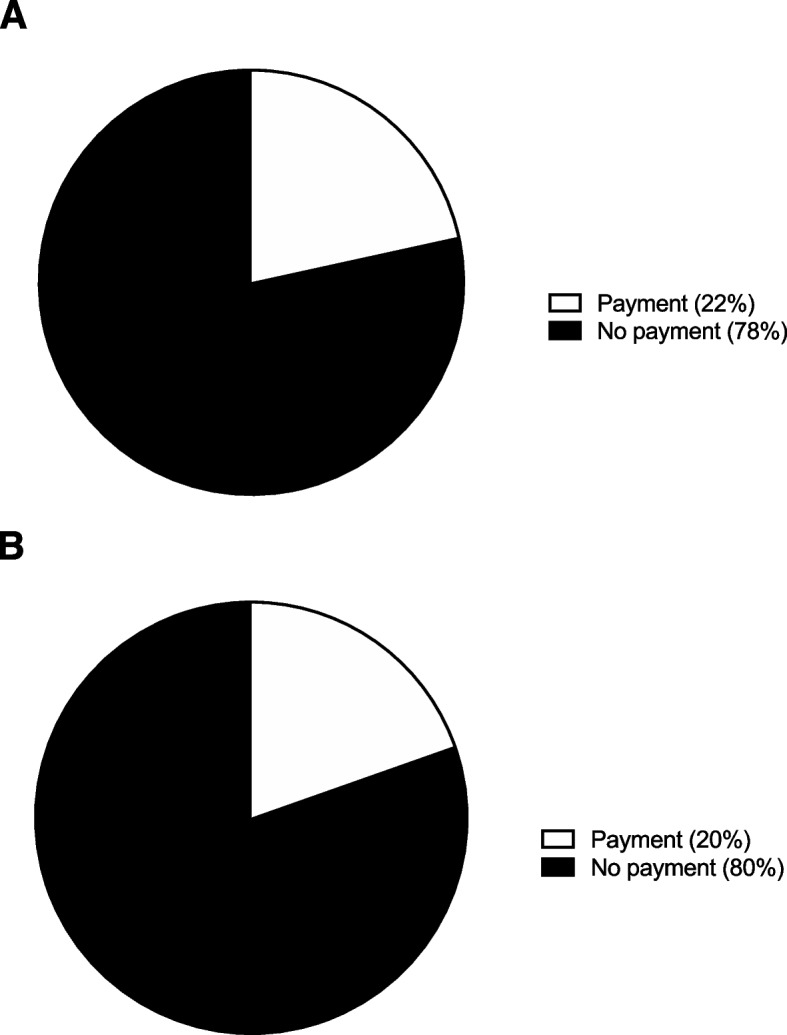
Telephone follow-up. **A** The percentage of participants indicating their willingness to pay for their two-week follow-up visit to be conducted over the telephone. **B** The percentage of participants indicating their willingness to pay for their six-week follow-up visit to be conducted over the telephone

A majority, 61 patients (60%), would not pay to see the physician instead of another provider at the two-week follow-up. However, 41 patients (40%) would pay a mean $759 ± 1,276 to see the physician instead of another provider at the two-week follow-up (Fig. 8A). A majority 57 patients (56%) would not pay to see the physician instead of another provider at the six-week follow-up. However, 45 patients (44%) would pay a mean $813 ± 1,337 to see the physician instead of another provider at the six-week follow-up (Fig. 8B). Additionally, a large majority, 80 patients (78%), would not pay to conduct their two-week follow-up visit over the phone. However, 22 patients (22%) would pay a mean $426 ± 666 to conduct their two-week follow-up visit over the phone (Fig. 9A). A large majority, 82 patients (80%), would not pay to conduct their six-week follow-up visit over the phone. However, 20 patients (20%) would pay a mean $485 ± 697 to conduct their six-week follow-up visit over the phone (Fig. 9B).

### Post-operative patient experience

Over half (51%) of the patients did not miss any work due to surgery. However, one-third needed to take more than 6 weeks off of work (Table 2). From the percentage patients who worked, 17 patients (17%) received an income through temporary disability when they were not working due to their surgery. Finally, 12 patients (12%) received an income through their employer as paid time off, while another 12 patients (12%) lost money as they did not receive any supplemental income after surgery (Figs. 7, 8 and 9).

**Table 2:** Postoperative experience of the patient and caretaker

Postoperative Experience	Response (*n* = 102)
How many days off of work did you have to take due to your surgery?	
0 days	52 (51%)
1–3 days	3 (3%)
4–7 days	2 (2%)
8–14 days	1 (1%)
15–21 days	5 (5%)
22–42 days	6 (6%)
Greater than 6 weeks	33 (33%)
Who helped take care of you after surgery (caretaker)?	
My spouse/significant other	38 (37%)
My mother/father	1 (1%)
My sibling	6 (6%)
My child	14 (14%)
A friend	8 (8%)
Other	33 (33%)
No one	2 (2%)
Did you still get paid while you were not working because of your surgery?	
My employer (paid time off)	12 (12%)
Temporary Disability	17 (17%)
Work insurance	2 (2%)
No one, I lost money	12 (12%)
No one, I do not work	55 (54%)
How many days off of work did your caretaker have to take?	
0 days	66 (65%)
1–3 days	7 (7%)
4–7 days	11 (11%)
8–14 days	4 (4%)
15–21 days	7 (7%)
22–42 days	3 (3%)
Greater than 6 weeks	4 (4%)

### Post-operative caretaker experience

A significant other or child provided assistance care for over half (51%) the patients after the surgery (Table 2). Another 33 patients (33%) stated they received post-operative care from someone designated as “other.” A majority of caretakers (65%) did not take time off of work to care for the patients after the surgery. 11 caretakers (11%) took less than seven days off of work to care for the patient after the surgery. Only 4% of caretakers (4%) needed to take more than 6 weeks off work.

### Anticoagulation

Overall, a significant number of patients preferred not to use anticoagulation in total joint arthroplasty (*p* = 0.019). However, value attributed to avoiding a specific method of anticoagulation was found to be not significant (*p* = 0.507, Table 3). A large majority, 74 patients (73%), would not pay to avoid having their blood drawn for anticoagulation every 2 to 4 days. The remaining patients (27%) would pay a mean $1,145 ± 1,705 to avoid having their blood drawn every 2 to 4 days. A large majority, 70 patients (73%), would not pay to avoid having daily injections to dose their medication. The remaining 32 patients (31%) would pay a mean $1,166 ± 1,711 to avoid daily injections. A large majority, 84 patients (82%), would not pay to avoid taking medication via pills. The remaining 18 patients (18%) would pay a mean $701 ± 1,131 to avoid taking pills. A large majority, 86 patients (84%), would not pay to avoid wearing leg compression devices. The remaining 16 patients (16%) would pay a mean $261 ± 361 to avoid wearing leg compression devices.

**Table 3:** Patient perception of route of administration of each type of anticoagulant used in total joint arthroplasty

Route of Anticoagulant	Prefer to Not Use*	Value to Avoid**
Oral with blood draw	28 (27%)	$322 ± 1,032
Injection	32 (31%)	$373 ± 1,103
Oral only	18 (18%)	$129 ± 458
Wearable	16 (16%)	$43 ± 173

^*^Pearson, *p* = 0.019 and χ^2^ = 9.90

^**^ANOVA, *F* = 0.9228 and *p* = 0.507

## Discussion

Overall, the study determined the metrics prioritized by hospitals and surgeons are not important to most patients when evaluating their care. With the standard incision size for a joint replacement is 12 cm, majority of the patients were not willing to pay more for a smaller incision size. A rationale for performing a smaller incision to access the joint reduces bacterial infections since a smaller soft tissue area is exposed during the surgery. Furthermore, a less invasive incision reduces the disruption of the quadricep muscle potentially accelerating post-operative rehabilitation [7]. Interestingly, the explanation for this advantage was not primarily for the benefit of the patient rather a necessary adjustment after the Affordable Care Act significantly reduced the approved number of outpatient visits to surgeons [7]. Another factor used to evaluate patient care is the time spent in the hospital with the standard being two days after surgery. From the study, majority of patients would not pay to extend their hospital stay, and patients would not need to be compensated to shorten their hospital stay. For primary hip replacements the average length of stay decreased from 9.1 days in 1991–1992 to 3.7 in 2007–2008 [8]. The 60% change over an 18-year period can be attributed to the development of newer technologies and methodologies to quicken patient recovery; however, the interests of the hospital system also contribute the decrease [8]. The adverse effect of shortening the hospital stay for the patient resulted in an increased 30-day all cause readmission rate from 5.9% in 1991–1992 to 8.5% in 2007–2008 [8].

The recovery after a total joint arthroplasty dictates the effectiveness of the surgery with standard of care being discharging patients to return home with family postoperatively. In the study, majority of patients would not pay to be discharged to a home health aide, physical therapy, or a skilled nursing facility. Therefore, the study participants indicated they would prefer to be discharged to their home. However, a previous study determined 82% of the 138,842 patients undergoing total hip arthroplasties and 79% of the 329,233 patients undergoing total knee arthroplasties were not discharged to their home [9]. Furthermore, another study determined patients discharged home demonstrated similar degrees of functional improvement as the patients discharged to a skilled nursing facility [10]. The patients discharged home were, also, no more likely to die 30 days after surgery [10, 11]. Taken together, patients are wanting to be discharged home; however, usually patients are discharged to another location such as a skilled nursing facility even though the recovery of patients in their home is similar to the patients in a nursing facility or rehabilitation center.

As important as recovery, patient follow-up visits are essential during the overall care for total joint replacement. These visits do not require the presence of the physician and subsequently the patients may meet another provider during their visits. In this study, patients would not pay additionally to meet with the surgeon and were satisfied with the standard follow-up visit with the nurse practitioner. Previous studies have determined the quality of care is maintained between a nurse practitioner and physician during follow-up visits. Kolb et al., found nurse practitioner follow-up reduced chemotherapy-related nephrotic syndrome prevalence and severity [12].

Furthermore, 80% of the patients would not pay for telemedicine follow-up visits. Interestingly, a previous study has found majority of the participants preferred to eliminate preferred office visits after a total joint replacement citing loss of wages and time [13]. The use of telemedicine is an effective cost reduction model for hospital systems when providing care. However, from this study, it is apparent patients do not want to conduct their medical care through telephone. Therefore, patients, physicians, and hospitals need to find a solution where patients receive the healthcare experience they require, while effectively utilizing the physician’s time and reducing hospital costs.

A major concern during total joint arthroplasty is the formation of blood clots; therefore, patients are administered an anticoagulation therapy for prophylaxis treatment. In this study, multiple options for anticoagulation treatment were provided ranging from injections to leg compressions. The participants did not indicate a strong preference towards one specific treatment modality. They would not pay more to reduce the number of daily blood draws, prevent injections or pills as the mode of their medication, or wear leg compressions. The variability found in this study is highlighted in a systematic review which analyzed the data from 48 different studies [14]. The conclusions drawn from the review determined patient values for choosing a particular anticoagulation therapy were highly variable [14]. Factors which determined the patient’s preference for the mode of anticoagulation therapy was based on previous experiences and health outcomes of the treatment method [14, 15]. Furthermore, a decision analysis was constructed for perioperative oral anticoagulants resulting in useful information for providers such as stratifying patient risk depending on their risk of stroke and previous cardiac health history [16]. However, the study did not include patients in the discussion of the decision analysis.

## Conclusions

This study determined values important for surgeons and hospital system did not align with the values patients deemed important during a total joint replacement. For example, patients prefer to be discharged to their home; however, most patients are being discharged to home health aides or skilled nursing facilities. Also, with increasing importance placed upon metrics to determine the overall efficiency and aptitude of a hospital, there is a greater effort to streamline aspects of the procedure. Through this study, the results determined patients do not want to participant in telemedicine; however, from the perspective of the hospital system, telemedicine would increase efficiency in interfacing with more patients. These disconnects in the entitlements patients expect from their care and the perceived entitlements, by the hospital, patients are actually receiving as care can be solved by including patients in the discussions with physicians and hospital systems. The continual balance between providing patients optimal care without spending excessive amounts of hospital resources can be maintained once the surgeon determines who they are aligning themselves with the patient or the hospital system. The pivotal role of the surgeon with these discussions will navigate the direction of medical care as patient-centered or system-centered.

## Supplementary Information

**Additional file 1.**

## Data Availability

The datasets used and/or analyzed during the current study are available from the corresponding author on reasonable request.
