# Supplementary material for: Lack of alignment between orthopaedic surgeon priorities and patient expectations in total joint arthroplasty
Source: Patient Saf Surg. 2023 Jun 29;17:17. doi: 10.1186/s13037-023-00365-w (PMC10308647; doi:10.1186/s13037-023-00365-w)
Supplement: Supplementary file 1 — Additional file 1. [file 13037_2023_365_MOESM1_ESM.pdf]

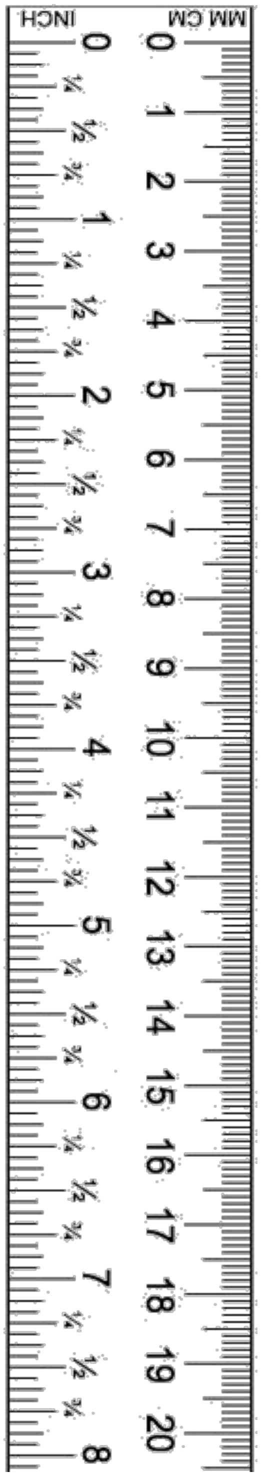

1. The standard incision size for a joint replacement is about 12 centimeters (cm). How much **more** money would you be willing to pay to have a **smaller** incision?

Size of Incision	Out of Pocket Payment
8 cm incision	$0<br>$1-$50: Amount $______<br>$51-250: Amount $______<br>$251-1000: Amount $_____<br>$1001-5000: Amount $_____<br>Over $5000: Amount $______

2. The standard incision size for a joint replacement is 12 centimeters (cm). How much **less** money would you be willing to pay to have a **bigger** incision?

Size of Incision	Out of Pocket Payment
16 cm	$0<br>$1-$50: Amount $______<br>$51-250: Amount $______<br>$251-1000: Amount $_____<br>$1001-5000: Amount $_____<br>Over $5000: Amount $______

3. The standard number of days in the hospital after surgery is 2 days. How much **more** money would you be willing to pay to stay **more** days?

Days in Hospital	Out of Pocket Payment
3 days	$0<br>$1-$50: Amount $______<br>$51-250: Amount $______<br>$251-1000: Amount $_____<br>$1001-5000: Amount $_____<br>Over $5000: Amount $______

4. The standard number of days in the hospital after surgery is 2 days. How much **less** money would you be willing to pay to stay **fewer** days?

Days in hospital	Out of Pocket Payment
1 day	$0<br>$1-$50: Amount $______<br>$51-250: Amount $______<br>$251-1000: Amount $_____<br>$1001-5000: Amount $_____<br>Over $5000: Amount $______

5. The standard return to activity time is 3 months. How much **more** money would you be willing to pay to return to your activities **earlier**?

Number of Weeks Earlier	Out of Pocket Payment
2 weeks	$0<br>$1-$50: Amount $______<br>$51-250: Amount $______<br>$251-1000: Amount $_____<br>$1001-5000: Amount $_____<br>Over $5000: Amount $______
4 weeks	$0<br>$1-$50: Amount $______<br>$51-250: Amount $______<br>$251-1000: Amount $_____<br>$1001-5000: Amount $_____<br>Over $5000: Amount $______

6. The standard discharge procedure after surgery is to return home with family. How much **more** money would you pay for an **alternate option**?

Alternate Option	Out of Pocket Payment
Home Health Aid	$0<br>$1-$50: Amount $______<br>$51-250: Amount $______<br>$251-1000: Amount $_____<br>$1001-5000: Amount $_____<br>Over $5000: Amount $______

7. The standard procedure after surgery is to have physical therapy (PT). How much could we **pay** you to **not have PT**?

Alternate Option	Your Paid
Self-guided PT	$0<br>$1-$50: Amount $______<br>$51-250: Amount $______<br>$251-1000: Amount $_____<br>$1001-5000: Amount $_____<br>Over $5000: Amount $______

8. Assuming you were medically cleared to discharge home after your joint replacement, how much would you be willing to pay to be discharged to a skilled nursing facility instead?

Alternate Option	Out of Pocket Payment
SNF instead of Home	$0<br>$1-$50: Amount $______<br>$51-250: Amount $______<br>$251-1000: Amount $_____<br>$1001-5000: Amount $_____<br>Over $5000: Amount $______

9. You are likely to see nurse practitioner at your 2 weeks and 6 weeks follow-up visits. How much **more** money you would pay to **see Dr. Amanatullah on each visit instead**?

Alternate Option	Out of Pocket Payment
2 Weeks with Dr. A	$0<br>$1-$50: Amount $______<br>$51-250: Amount $______<br>$251-1000: Amount $_____<br>$1001-5000: Amount $_____<br>Over $5000: Amount $______
6 Weeks with Dr. A	$0<br>$1-$50: Amount $______<br>$51-250: Amount $______<br>$251-1000: Amount $_____<br>$1001-5000: Amount $_____<br>Over $5000: Amount $______

10. You are most likely to see nurse practitioner **in the office** at your 2 weeks and 6 weeks follow-up. How much **more** money you would pay to **visit over the phone and skip the office visit**?

Alternate Option	Out of Pocket Payment
2 Weeks Over Phone	$0<br>$1-$50: Amount $______<br>$51-250: Amount $______<br>$251-1000: Amount $_____<br>$1001-5000: Amount $_____<br>Over $5000: Amount $______
6 Weeks Over Phone	$0<br>$1-$50: Amount $______<br>$51-250: Amount $______<br>$251-1000: Amount $_____<br>$1001-5000: Amount $_____<br>Over $5000: Amount $______

11. In joint replacement surgery, your joint surface will be replaced by an implant. How **much** more money you would pay to **pick your implants**.

Alternate Option	Out of pocket payment
Pick your Implant	$0<br>$1-$50: Amount $______<br>$51-250: Amount $______<br>$251-1000: Amount $_____<br>$1001-5000: Amount $_____<br>Over $5000: Amount $______

12. In a joint replacement surgery, different methods are used prevent a blood clot. How much **more** money would you pay to **NOT have you blood drawn with a needle every 2 to 4 days to dose the chosen medication**?

Alternate Option	Out of Pocket Payment
No Blood Draw	$0<br>$1-$50: Amount $______<br>$51-250: Amount $______<br>$251-1000: Amount $_____<br>$1001-5000: Amount $_____<br>Over $5000: Amount $______

13. In a joint replacement surgery, different methods are used prevent a blood clot. How much **more** money would you pay to **NOT have a daily injection to dose the chosen medication**?

Alternate Option	Out of Pocket Payment
No Injection	$0<br>$1-$50: Amount $______<br>$51-250: Amount $______<br>$251-1000: Amount $_____<br>$1001-5000: Amount $_____<br>Over $5000: Amount $______

14. In a joint replacement surgery, different methods are used prevent a blood clot. How much **more** money would you pay to **NOT have to take a pill to dose the chosen medication**?

Alternate Option	Out of Pocket Payment
No Pills	$0<br>$1-$50: Amount $______<br>$51-250: Amount $______<br>$251-1000: Amount $_____<br>$1001-5000: Amount $_____<br>Over $5000: Amount $______

15. In a joint replacement surgery, leg compression may be used prevent a blood clot. How much **more** money would you pay to **NOT have to wear leg compression devices all day long**?

Alternate Option	Out of Pocket Payment
No Compression	$0<br>$1-$50: Amount $______<br>$51-250: Amount $______<br>$251-1000: Amount $_____<br>$1001-5000: Amount $_____<br>Over $5000: Amount $______
